# Serious complications after button battery ingestion in children

**DOI:** 10.1007/s00431-018-3154-6

**Published:** 2018-05-02

**Authors:** Hilde Krom, Margot Visser, Jessie M. Hulst, Victorien M. Wolters, Anita M. Van den Neucker, Tim de Meij, Hubert P. J. van der Doef, Obbe F. Norbruis, Marc A. Benninga, Margot J. M. Smit, Angelika Kindermann

**Affiliations:** 1 Emma Children’s Hospital, Academic Medical Center, Amsterdam, Netherlands; 2 Willem-Alexander Children’s Hospital, Leiden University Medical Center, Leiden, Netherlands; 3 Erasmus Medical Center, Sophia Children’s Hospital, Rotterdam, Netherlands; 4 Wilhelmina Children’s Hospital, University Medical Center Utrecht, Utrecht, Netherlands; 5 Maastricht University Medical Center +, Maastricht, The Netherlands; 6 VU University Medical Center, Amsterdam, Netherlands; 7 University of Groningen, University Medical Center Groningen, Groningen, Netherlands; 8 Isala Zwolle, Zwolle, Netherlands; 9 Juliana Children’s Hospital, Haga Teaching Hospital, Den Haag, Netherlands

**Keywords:** Button battery, Foreign body, Ingestion, Complications, Pediatrics

## Abstract

Serious and fatal complications after button battery ingestion are increasing worldwide. The aim of this study is to describe serious complications after battery ingestion in children in the Netherlands.

All pediatric gastroenterologists in the Netherlands performing upper endoscopies were asked to report all serious complications after battery ingestion in children (0–18 years) between 2008 and 2016 retrospectively.

Sixteen serious complications were reported: death after massive bleeding through esophageal-aortal fistula (*n* = 1), esophageal-tracheal fistula (*n* = 5), stenosis after (suspected) perforation and mediastinitis (*n* = 5), (suspected) perforation and mediastinitis (*n* = 3), vocal cord paralysis (*n* = 1), and required reintubation for dyspnea and stridor (*n* = 1). The median time interval between ingestion and presentation was 5 (IQR 2–258) h. All children were ≤ 5 (median 1.4; IQR 0.9–2.1) years. Vomiting (31.3%), swallowing/feeding problems (31.3%), and fever (31.3%) were the most common presenting symptoms; however, 18.8% of the patients were asymptomatic (*n* = 1 missing). All batteries were button batteries (75% ≥ 20 mm; 18.8% < 20 mm; *n* = 1 missing). The batteries were removed by esophagogastroduodenoscopy (50%) and rigid endoscopy (37.5%) or surgically (12.5%).

*Conclusion*: Sixteen serious complications occurred after small and large button batteries ingestion between 2008 and 2016 in both symptomatic and asymptomatic children in the Netherlands. Therefore, immediate intervention after (suspected) button battery ingestion is required.

**Table:** 

**What is Known:** *• Button battery ingestion may result in serious and fatal complications.* *• Serious and fatal complications after button battery ingestion are increasing worldwide.*	
**What is New:** *• Sixteen serious complications after button battery ingestion occurred during 2008–2016 in children in the Netherlands.* *• Serious complications were also caused by small batteries (< 20 mm) in the Netherlands and also occurred in asymptomatic Dutch children.*

## Introduction

Children often put toys and other foreign bodies in their mouth and sometimes even swallow them. Older children with mental disabilities are also at risk for foreign body ingestion. Most of the time, small ingested foreign bodies pass the gastrointestinal tract spontaneously without complications. They can, however, cause serious damage especially when they get stuck in the esophagus [9, 14]. Button batteries in particular are able to cause serious esophageal injuries within 2 h after ingestion [10]. The number of serious complications and mortalities caused by button battery ingestions has been risen in recent years [11]. The most common symptoms after button battery ingestion are dysphagia, coughing, fever, and vomiting [2]. These symptoms are nonspecific and can also be seen in many other diseases [7].

Esophageal-tracheal fistula, esophageal perforation, esophageal stenosis, vocal cord paralysis, mediastinitis, pneumothorax, aspiration pneumonia, empyema, lung abscess, spondylodiscitis, esophageal-aortal fistula, and mortality due to respiratory and circulatory failure are serious complications which can occur after button battery ingestion. Serious complications especially develop in children. A study from the USA showed that 39/41 serious complications reported to the National Battery Ingestion Hotline occurred in children aged 0–19 years, and two reported deaths were both in children [10].

The aim of our study was to analyze the number and kind of serious complications after button battery ingestion in children in the Netherlands.

## Materials and methods

All esophagogastroduodenoscopy-performing pediatricians were asked by the Dutch Society of Pediatric Gastroenterology, Hepatology and Nutrition to complete a case report form for all children aged 0–18 years with serious complications after the ingestion of a battery during the period 2008–2016 retrospectively. They were asked to register the time interval between ingestion and presentation in the hospital (not based on symptoms; ingestion was witnessed or the battery was missed by the parents since a certain time), symptoms after ingestion, type and the measured diameter of the battery, location of impaction, intervention, occurrence of complications, time of hospitalization, and long-term damage based on data in the medical records of the patients.

We included all serious complications, which were defined as the development of a fistula, perforation, mediastinitis, stenosis, and mortality. We excluded patients without complications and patients with only short-term problems and/or complications, such as esophageal erosion or ulceration without perforation.

The Medical Ethics Committee of the Academic Medical Center in Amsterdam, the Netherlands, confirmed that the Medical Research Involving Human Subjects Act does not apply to the present study.

## Results

In total, 16 cases of serious complications after button battery ingestion were reported by esophagogastroduodenoscopy-performing pediatricians from six tertiary care hospitals and two large regional general hospitals. All ingested batteries were button batteries. All children were ≤ 5 years of age (median 1.4; IQR 0.9–2.1). The time interval between ingestion of the button battery and arrival in the hospital was ≤ 5 h in 56% (*n* = 9/16) varying from 1 h to 3 months (median 5.0; IQR 2.1–258.0). The symptoms after button battery ingestion at first presentation in the hospital are shown in Table 1. Vomiting, swallowing and feeding problems, and fever were the most common symptoms. In 75% of the children, the button battery was located in the upper part and in 25% in the middle part of the esophagus. The diameter of the button battery was ≥ 20 mm (*n* = 12/15; *n* = 1 unknown) in 80% of the patients. The three smaller (< 20 mm) button batteries, which were ingested and led to serious complications in children aged 0.9, 2.4, and 1.4 years, had diameters of 13, 15, and 17 mm respectively. The button batteries were removed by flexible esophagogastroduodenoscopy by pediatric gastroenterologists (50%) and rigid endoscopy by ear nose throat (ENT) specialists (37.5%) or surgically (thoracotomy, 12.5%). Thirteen patients (81.3%) received antibiotics after removal. The serious complications after button battery ingestion are shown in Table 2 and further explained below. A summary of each case is depicted in Table 3.

**Table 1:** Symptoms at first presentation after button battery ingestion

Symptoms	*n* (%)^*^
Vomiting	5 (31.3)
Swallowing and feeding problems	5 (31.3)
Fever	5 (31.3)
Drooling	3 (18.8)
Coughing	3 (18.8)
Gagging	3 (18.8)
Weight loss	2 (12.5)
Dyspnea	1 (6.3)
Sore throat	1 (6.3)
Melaena	1 (6.3)
Diarrhea	1 (6.3)
Distended abdomen	1 (6.3)
Pain between scapulae	1 (6.3)
Drowsiness	1 (6.3)
Asymptomatic	3 (18.8)
Unknown	1 (6.3)

*Concerning patients may have multiple symptoms, the total does not equal *n* = 16 (100%)

**Table 2:** Serious complications after button battery ingestion

Complication	*n* (%)
Mortality	1 (6.3)
Esophago-tracheal fistula(e)	5 (31.1)
Stenosis after (suspicion of) perforation and mediastinitis	5 (31.1)
(Suspicion of) perforation and mediastinitis	3 (18.8)
Vocal cord paralysis	1 (6.3)
Reintubation due to dyspnea and stridor	1 (6.3)
Total	16 (100)

**Table 3:** Case summary

Case	Age (year)	Diameter battery (mm)	Time to presentation (h)	Symptoms at presentation	Death/EAF	ETF	Stenosis after perforation and mediastinitis	Perforation and mediastinitis	Vocal cord paralysis	RI	Other complications	Clinical course
1	1.81	20	336	Sore throat, vomiting, swallowing and feeding problems, fever	x						Death due to massive bleeding through EAF	Thoracotomy
2	0.76	20	16.5	Coughing, drooling, vomiting		x					Mediastinitis	Rigid endoscopy, gastrostomy, tracheal cannula, stent placements, long-term admittance intensive care. Healing trachea with trachea malacie
3	1.37	21	288	Swallowing and feeding problems, coughing, weight loss, fever		x	x					Gastroscopy, spontaneous healing esophago-tracheal fistula, multiple stent placements, temporarily gastrostomy, admittance intensive care
4	1.38	17	588	Fever, coughing, swallowing and feeding problems		x		x		x	Full left lung atelectasis, aortic bleeding, arteria spinalis anterior syndrome with paralysis both legs	Thoracotomy, ECMO, esophagotomy, gastrostomy, resuscitation, re-thoracotomy twice, aortic stent, jejunostomy, second gastrostomy, esophageal drain
5	0.75	20	168	Diarrhea, distended abdomen, vomiting, fever		x				x		Gastroscopy, ECMO, reintubation, partial tracheal resection, mucus neck fistula
6	1.75	20	1	Asymptomatic		x				x		Gastroscopy, readmittance 6 days after removal because of respiratory insufficiency as a consequence of multiple fistulas. Surgical correction should take place in the future
7	5.04	21	2160	Swallowing and feeding problems, weight loss, pain between scapulae			x					Gastroscopy, gastrostomy, partial esophageal resection, central venous entrances, multiple dynamic stent placements and dilatations
8	2.23	24.3	2	Asymptomatic			x			x		Gastroscopy, regastroscopy and rigid endoscopy, intubation
9	2.43	15	3.5	Vomiting, drowsiness			x					Gastroscopy
10	0.90	13	1.5	Drooling			x					Rigid endoscopy/Magill, multiple dilatations
11	1.41	U	2.5	Gagging			x					Rigid endoscopy, multiple dilatations
12	0.58	20	1	Asymptomatic				x				Gastroscopy and rigid endoscopy
13	1.03	20	72	Swallowing and feeding problems, melaena, fever				x				Gastroscopy, swallowing problems for 2 months
14	4.50	21	5	Gagging				x		x		Rigid endoscopy
15	1.86	22.3	5	Unknown					x			Rigid endoscopy/laryngoscopy, gastroscopy, trachea cannula, decanulation and closure tracheostomy after 1.5 years
16	0.91	24.3	2.5	Drooling, dyspnoea, vomiting, gagging						x		Gastroscopy together with ENT, swallowing and feeding problems persisted for 4 months

*U* unknown, *EAF* esophago-aortal fistula, *ETF* esophago-tracheal fistula, *RI* respiratory insufficiency

### Mortality

One patient, 21 months old, presented 2 weeks after button battery ingestion with a sore throat, vomiting, less appetite, dysphagia, and fever. He died during thoracotomy, due to a massive bleeding caused by an esophageal-aortal fistula.

### Esophageal-tracheal fistula(e)

Five patients developed esophago-tracheal fistulae resulting in long-term damage. The time interval between ingestion of the button battery and presentation in the hospital varied (1 h, 1 day, and 1, 1.5, and 3.5 weeks).

One patient developed an esophago-tracheal fistula and a small fistula to the mediastinum resulting in a pneumomediastinum 1 week after removal of the button battery, followed by multiple stent placements, a gastrostomy, and a tracheal cannula.

The second patient developed an esophago-tracheal fistula, for which multiple stent placements and a gastrostomy were necessary. The esophago-tracheal fistula finally closed spontaneously, complicated with development of a stenosis.

The third patient underwent a thoracotomy immediately, due to the location of the battery (1–2 mm from the aorta) at CT. The aortic arch was exposed and the button battery was removed by esophagotomy. After placement of a gastrostomy, the patient developed an esophago-bronchial fistula and needed resuscitation by decanulation of the extracorporeal membrane oxygenation (ECMO). Eight months after battery ingestion, the patient developed a virus-induced respiratory insufficiency, mediastinitis, and full left lung atelectasis. During the following months, the patient underwent re-thoracotomy twice with fistulectomy of the esophago-tracheal fistula. During one re-thoracotomy, a massive bleeding of the aorta descendens developed, requiring stenting. Subsequently, the patient developed an arteria spinalis anterior syndrome with paralysis of both legs. After this, the patient developed an esophageal fistula in the neck; therefore, a jejunostomy, a second gastrostomy catheter, and a drain in the distal esophagus for draining were placed.

The fourth patient had an esophago-tracheal fistula, which was closed while on ECMO. However, in a later stage, the patient had to be re-intubated because of respiratory insufficiency, and a partial tracheal resection was necessary because of a recurrent esophago-tracheal fistula. After this resection, the patient got a mucus neck fistula to drain the saliva, and to let the esophageal ulcer heal consequently.

The button battery of the fifth patient was removed within 4 h after ingestion. This patient was readmitted 6 days after removal because of respiratory insufficiency as a consequence of multiple fistulas from the esophagus to the trachea and lung. During data collection for the present study, a surgical correction was planned but not yet been performed.

### Stenosis after (suspicion of) perforation and mediastinitis

Five patients developed an esophageal stenosis. The first patient developed a pinpoint stenosis and perforation after removal of the battery 3 months after ingestion. This patient underwent a partial esophageal resection, gastrostomy placement, and multiple esophageal dilatations. After 2 years, the patient was symptom free.

The second patient came to the hospital 2 h after ingestion, but without any symptoms. However, this patient developed respiratory insufficiency 1 day later, after self-removal of a nasogastric tube. During re-endoscopy by the ENT specialist and pediatric gastroenterologist, mild larynx edema and a long circular lesion were found in the esophagus with a suspicion of mediastinitis. After 3 months, the patient did not show abnormalities anymore.

The third patient, who arrived at the hospital within 3.5 h after ingestion, showed serious necrosis during removal of the small button battery (diameter 15 mm). One day after removal, the patient developed fever, and a mediastinitis was suspected. During re-endoscopy, a pinpoint stenosis of the esophagus was found. Due to the stenosis, feeding problems persisted for several months.

The fourth and fifth patients with stenosis after button battery ingestion required multiple dilatations. The fourth patient arrived in the hospital within 1.5 h after button battery (diameter 13 mm) ingestion and needed three dilatations of the esophagus. The fifth patient arrived in the hospital within 2.5 h after button battery (diameter unknown) ingestion and needed several dilatations within a period of 2.5 months.

### (Suspicion of) esophageal perforation and mediastinitis

Three patients were suspected for an esophageal perforation. One of them arrived in the hospital within an hour after button battery ingestion without any symptoms, but developed a pneumomediastinum. Another patient showed fever at presentation in the hospital and was suspected for perforation. The child had swallowing problems/dysphagia during 2 months. The third patient was suspected for a perforation of a circular lesion in the esophagus.

### Vocal cord paralysis

One patient developed a respiratory insufficiency based on a double-sided vocal cord paralysis, necessitating tube feeding and tracheostomy. After a period of 1.5 year, the patient was decanulated, the tracheostomy was closed, and tube feeding was ceased.

### Reintubation for dyspnea and stridor

One patient was ventilated at the Pediatric Intensive Care Unit, because of severe dyspnea and stridor, probably provoked by manipulation during removal of the button battery. Swallowing problems persisted for 4 months.

## Discussion

The present study describes 16 serious complications after button battery ingestion in children during an 8-year period in the Netherlands. Death after massive bleeding through esophageal-aortal fistula, esophageal-tracheal fistula, stenosis after (suspected) perforation and mediastinitis, (suspected) perforation and mediastinitis, vocal cord paralysis, and the need for reintubation because of dyspnea and stridor were found in these young children. Serious complications were caused by large (≥ 20 mm) as well as small batteries (< 20 mm) and occurred in symptomatic as well as in asymptomatic children.

It is known that ingestion of a button battery can result in serious complications, especially when it is impacted in the esophagus. Batteries can cause damage due to three mechanisms: direct pressure on the mucous membrane (pressure necrosis), leakage of battery contents (chemical damage), and electrical current generated by contact of the battery poles against the mucus membrane in the esophagus (electrical damage) [6, 8]. The third mechanism is thought to cause the most damage due to hydrolysis, which leads to hydroxide at the negative pole of the battery, which results in a high pH. The mucus membrane touching the negative pole of the battery will be damaged the most [5, 11]. The new lithium 3-V button batteries are even more dangerous than the older 1.5-V button batteries. The possibility of damage after ingestion of an unused button battery is larger than after a used one, due to their discharge state. However, even (almost) spent button batteries are able to cause damage [10].

Litovitz et al. showed that the diameter of an ingested button battery is an important predictor for the development of serious complications. In this study from the USA, the diameter of the battery was ≥ 20 mm in 93.9% (31/33) of the children with serious complications and/or mortality after battery ingestion. Complications were most severe in children < 4 years of age who ingested a large lithium button battery (≥ 20 mm). The incidence of large button battery (≥ 20 mm) ingestion is increasing during recent years (from 1% in 1990 up to 18% in 2008) [10, 11]. These batteries are used for household appliances, remote controllers, and toys [10, 11]. Our study in the Netherlands, however, showed that even batteries with small diameters (< 20 mm) can lead to serious complications.

Symptoms after button battery ingestion are nonspecific and can also be seen in other diseases, such as viral infections [7]. In a review, symptoms of 188 children with a battery in the mouth, esophagus, or stomach were described. The most common symptoms were dysphagia (30.2%), coughing (26.4%), fever (26.4%), and vomiting (17.3%) [2]. This is in accordance with the observations in our population, in which vomiting (33.3%), swallowing and/or feeding problems (27.8%), and fever (27.8%) were the most common symptoms. When the ingestion of the button battery is not witnessed and, therefore, not mentioned in the history by the parents and/or caretakers, it can be difficult to recognize the correct diagnosis due to the nonspecific character of the symptoms after button battery ingestion. A delay in diagnosis and treatment can increase the likelihood of serious complications consequently [7].

Serious complications after button battery ingestion in the Netherlands occurred both in children who arrived in the hospital within 1 h and in children who arrived with delay. This finding is in accordance with previous literature [3, 5, 10]. Animal studies have shown that necrosis of the lamina propria in the esophagus can develop within 15 min and can expand to the outer muscular layer of the esophagus within 30 min after button battery ingestion [13]. Serious complications after the ingestion of button batteries are described in children both with removal of the battery within a short period after ingestion (2–2.5 h) and with removal after a longer period (3 months) [3, 5, 10]. Even after removal, complications can still develop. A case of a 2-year-old girl was published, who died as a consequence of a massive bleeding through an esophageal-aortal fistula 18 days after button battery removal [1]. In our study, also one child died due to massive bleeding through an esophageal-aortal fistula 2 weeks after ingestion. Esophageal-aortal fistulae are the most common cause of death after ingestion of a button battery [1, 10].

Like in our study, esophageal-tracheal fistula, esophageal perforation, esophageal stenosis, vocal cord paralysis, mediastinitis, pneumothorax, aspiration pneumonia, empyema, lung abscess, spondylodiscitis, esophageal-aortal fistula, and mortality due to respiratory and circulatory failure are the most frequently described complications [10]. Esophageal-tracheal fistulae can also develop several days after button battery removal, and a stenosis weeks to months after ingestion [10]. A large American study analyzing 8468 battery ingestions showed that the ingestion of batteries with a diameter of 20–25 mm led to serious complications or mortality in 12.6% of the children younger than 6 years of age [11].

National guidelines considering the treatment after button battery ingestion are lacking in the Netherlands, amongst other European countries. In 2015, the North American Society for Pediatric Gastroenterology, Hepatology, and Nutrition (NASPGHAN) published an international guideline considering the management of ingested foreign bodies in children [9]. The European Society for Paediatric Gastroenterology, Hepatology and Nutrition (ESGHAN) and European Society of Gastrointestinal Endoscopy (ESGE) followed with an international guideline in 2017 [14]. Both the NASPGHAN and ESPGHAN recommend to perform emergent (< 2 h) endoscopic removal for button batteries impacted in the esophagus in both symptomatic as asymptomatic children [9, 14]. The ESPGHAN suggests to remove button batteries in the stomach < 2 h if the child shows symptoms, has (suspected) anatomical pathology in the gastrointestinal tract, and/or has swallowed a magnet simultaneously with the button battery ingestion. Furthermore, the ESPGHAN guideline suggests to check button batteries > 20 mm present in the stomach radiographically, and if the battery is still in place after > 48 h to remove it. The ESPGHAN guideline describes their recommendations considering batteries to be based on low to moderate quality of evidence [14]. The NASPGHAN guideline recommends to consider removing gastric and small bowel button batteries endoscopically emergent when having symptoms, and urgently/within 24–48 h in children aged ≤ 5 years and if the battery is ≥ 20 mm. Button batteries are suggested to be removed by elective (> 24 h after ingestion) endoscopy when symptoms start in previously asymptomatic patients or when the battery is not moving on serial X-rays. The guideline of the National Battery Ingestion Hotline recommends to remove batteries in the stomach when symptoms appear, and in children < 6 years without symptoms if batteries ≥ 15 mm are still in place in the stomach 4 days after ingestion in children (https://www.poison.org/battery/guideline). Furthermore, the NASPGHAN guideline suggests to consider CT angiography or chest MRI if any esophageal injury is present after removal of the battery from the esophagus [9]. CT or MRI scans can also be considered when esophageal damage is present while the battery was in the stomach or beyond [9].

Considering the severity of the complications after button battery ingestion published in the present study and earlier published literature, and because the recommendations of international guidelines are mostly based on low quality of evidence literature, we emphasize the need for national guidelines recommending uniform country-specific policy after button battery ingestion in children. Based on earlier published international guidelines [9, 14], we encourage following course of actions. If a button battery ingestion is suspected, parents and/or caretakers should immediately contact a physician. Regardless of the diameter of the button battery, a radiography (from mouth to abdomen, biplane if necessary) should be performed even in an asymptomatic patient. If the button battery is located in the hypopharynx or esophagus, immediate removal is necessary, regardless of battery diameter, time interval between ingestion and arrival, and the presence of symptoms [4, 8, 12]. When the patient is bleeding actively or is clinically unstable, endoscopic removal should take place with surgery or cardiovascular surgeon being present [9]. If the battery already passed the esophagus, a pediatric gastroenterologist should be contacted, who may take the child’s age and symptoms and the diameter of the button battery into account in the decision for the appropriate course of action.

The Dutch Pediatric Society is working on a national guideline concerning the ingestion of foreign bodies at the moment, in which battery ingestion will be of importance as well. Furthermore, a nationwide Dutch registration system for all pediatricians started in January 2018 to register and analyze all battery ingestions in children < 0-18 years old. This will allow us to calculate the risk of (serious) complications after ingestion to assess potential risk factors.

### Limitations of the study

In this study, we aimed to analyze all serious complications after battery ingestions in children in the Netherlands during a long period. However, some limitations have to be considered. The battery diameter was measured. The imprint code would have been more reliable, but due to the retrospective design of the study, this was not available. Furthermore, we are aware of the possibility of missing patients, such as another 1-year-old boy who got publicity in the Dutch media, after he died due to a button battery ingestion during our collection period. This patient was not included, because he died before an esophagogastroduodenoscopy could have been performed. In addition, we might have missed patients, where batteries were removed by an otolaryngologist or adult gastroenterologist. On the other hand, in the Netherlands, it is very unlikely that this would happen without involving a pediatrician and pediatric gastroenterologist. Furthermore, we did not include less serious complications in the present study. However, even those children without or with minor complications are affected by negative consequences of ingestion (such as unpleasant complaints, the impact of admission to the hospital, anesthesia, esophagogastroduodenoscopy, and radiation exposure by imaging).

## Conclusion

The present study described 16 serious complications (including mortality) after button battery ingestion in young children of whom some showed several symptoms but others were completely asymptomatic. Serious complications developed both after a short and after a long period between ingestion and presentation. Even small batteries can cause serious complications. Herewith, we hope to raise awareness to the serious complications following button battery ingestion in children. We encourage incorporating the course of action after button battery ingestion in national guidelines.

## Abbreviations

CT scan: Computerized tomography scan
ECMO: Extracorporeal membrane oxygenation
IQR: Inter quartile range
